# Ecotoxicological Effects of Heavy Metals on Rice (*Oryza sativa* L.) Across Its Life Cycle and Health Risk Assessment in Soils Around Pb–Zn Mine

**DOI:** 10.3390/plants15010030

**Published:** 2025-12-21

**Authors:** Fangyu Hu, Baoyu Wang, Lingyan Zhang, Yue Wang, Jiaqi Sha, Jinhao Dong, Hewei Song, Jing An

**Affiliations:** 1 Institute of Applied Ecology, Chinese Academy of Sciences, Shenyang 110016, China; hufangyu@iae.ac.cn (F.H.); wangbaoyu2024@126.com (B.W.); zhangly@iae.ac.cn (L.Z.); 15114187525@163.com (Y.W.); shajiaqi77@163.com (J.S.); jhdong1999@163.com (J.D.); jlushw@126.com (H.S.); 2 University of Chinese Academy of Sciences, Beijing 100049, China

**Keywords:** Pb-Zn mine, heavy metals, rice, toxicity, health risk assessment

## Abstract

Agricultural soils surrounding mining areas are often polluted with heavy metals (HMs) due to long-term mining activities and high geological background values. In this study, we investigated the distribution and transport of Cu, Cr, Zn, Cd, Pb, and As in a soil–rice system near a century-old mining site, evaluated their toxic effects on rice (*Oryza sativa* L.) throughout the growth period, and assessed the associated health risks using the Nemerow index and potential ecological risk index. The results showed that HM contents in rice grown in contaminated soils were significantly higher than in the control. HMs mainly accumulated in roots, with the lowest contents in grains. Cd exhibited the highest enrichment capacity, with bioconcentration factors of 0.79, 1.04, and 1.95 at the tillering, heading, and maturity stages, respectively, and its accumulation increased with rice growth. Transport from stems to leaves was relatively strong. HM exposure significantly inhibited rice growth, reducing plant height, biomass, tiller number, and panicle emergence. In addition, oxidative stress indicators and antioxidant enzyme activities, as well as root amino acid exudation, were markedly altered under HM stress. According to soil–rice HM contents, the pollution level of agricultural soils reached a high class, with As, Pb, Cd, and Zn as the main contributors. The potential ecological risk reached a moderate level, with Cd identified as the dominant factor. Notably, the health risks to children were substantially higher than those to adults, and Monte Carlo simulation indicated a 100% probability of non-carcinogenic and carcinogenic risks for adults and children. The above results highlighting the urgent need for risk management in mining-affected regions.

## 1. Introduction

The mining and smelting industry have significantly contributed to the country’s economic growth [1]. However, metal processing, smelting, and other mining-related activities can generate significant amounts of waste gas, wastewater, and residues that may result in the pollution of the surrounding environment with heavy metals (HMs) [2,3]. It was reported that the contents of Hg, Cd, As, Cu, and Zn in soil samples around the Wanshan mercury mine mostly exceeded national standards [4]). Previous studies have found that Cu and Cd were the primary contaminants in the soil surrounding the Dexing copper mine, with concentrations exceeding the risk screening values for agricultural land contamination [5]. Among them, Pb-Zn mines are characterized by complex ore types and often accompanied by other heavy metal elements [6]. Therefore, the mining and smelting processes may result in more severe pollution of the surrounding soil. It has been reported that HMs such as Cd, Pb, Cr, Cu, Zn, and As in the soil of a Pb-Zn smelting area in Huize City exceeded Chinese environmental quality standards for soil [7]. Previous studies also found that Pb-Zn mining areas generally faced a serious problem of HM pollution, and the average concentrations of As, Cd, Cr, Cu, Hg, Ni, Pb and Zn had exceeded national standards [8]. HMs cannot be degraded after entering the soil and polluting nearby soil and rivers through surface runoff and groundwater. This results in an elevation of HM content within the vicinity of the mining site, particularly in the soil, leading to environmental pollution [9]. More importantly, HM pollution may endanger human health through the soil–crop–food pathway [10]. Therefore, it is imperative to investigate the potential health risks posed by HMs in agricultural soils near large mining areas to both ecosystems and human populations.

Paddy rice (*Oryza sativa* L.) is a primary global staple food [11,12]. Meanwhile, it has been demonstrated that rice exhibits a greater capacity for bioaccumulation and translocation of HMs compared to other grain crops, primarily due to its botanical characteristics and planting environment [13,14]. Therefore, rice is considered one of the most significant model plants for assessing the ecological health risks posed by HMs due to its widespread cultivation and high sensitivity to contaminants. After uptake by rice roots, HMs are translocated and accumulated in the rice grains [15]. For example, Cd is absorbed by rice roots, transported upward through the xylem, redistributed between stems and leaves, and transferred to the grains via the bast [16]. Previous studies found that Cd and Cr were detected in rice grains exceeding the national food contaminant limit standard in the core of Asia’s largest karst region, which increases the likelihood of human exposure to HMs and poses a potential risk to both ecosystem and human health [17]. The enrichment of HMs in rice and their accumulation in soil lead to significant ecotoxicological effects on rice throughout its growth cycle. It has been reported that the presence of HMs in crops may disrupt enzymes essential for normal crop metabolism and development, thereby impeding photosynthetic processes as well as water and nutrient uptake, ultimately inhibiting crop growth [18]. For example, excess Cd can affect the growth of maize plants and reduce the chlorophyll content in leaves [19]. Additionally, excess Zn in the soil significantly inhibits rice growth and causes plant chlorosis [20]. Currently, research is focused on the ability of rice to accumulate and translocate HMs during the maturity period, with less attention given to its changes during different growth periods in response to HM stress.

How to assess the health risk of HMs effectively and scientifically in agriculture soil-crop systems has been a topic of great attention for decades. The Nemerow index is one of the most commonly used contamination indexes for evaluating the level of heavy metal contamination in soil. Previous studies assessed the pollution of Pb-Zn mines in Huayuan county using Nemerow index and found that the Cd, Zn, and Ni in the paddy soil around the mining area were up to pollution level [1]. In addition, the potential ecological risk index (RI) is generally used for calculating the ecological risk of HMs in soil based on toxic response factors [21]. It has been reported that RI was used to assess the potential ecological risk of HMs in the Ningbo area and found that Hg was the most contaminated heavy metal in the soil, posing the highest ecological risk [22]. With the advancement of risk assessment methodologies, there is an increasing focus on constructing and implementing assessment models [21,23]. The non-carcinogenic and carcinogenic risk models are used to evaluate the health risks of HMs on humans. Many previous studies have reported the health risks of HMs, such as Pb damaging the human central nervous system, causing damage to human skin, and being highly carcinogenic, and Cd exposure leading to liver, skin, and other cancers [24,25]. Most previous studies have relied on field sampling. However, field samples are affected by many environmental factors, such as soil type, irrigation practices, fertilization regimes [26]. These factors introduce substantial uncertainty and make heavy metal accumulation and the resulting health risk assessments less controllable.

The Qingchengzi Pb-Zn mine is a key mining area in the northeast lead-zinc production base of China, boasting abundant mineral resources and complex ore types with a long history of exploitation. However, extensive and prolonged mining activities have resulted in environmental pollution that has affected the surrounding farmland. This study aims, through controlled pot experiments, to (1) evaluate the distribution and transport of HMs in soil–rice systems across the entire rice growth period; (2) investigate the toxic effects of HMs on rice from the tillering to maturity stages; and (3) assess the ecological and human health risks associated with HMs in agricultural soils surrounding mining areas, thereby providing insight into the impacts of mining activities on regional ecological health.

## 2. Results and Discussion

### 2.1. Absorption and Bioaccumulation of HMs in the Life-Cycle of Rice

#### 2.1.1. The Content of HMs in Different Parts of Rice at Critical Growth Periods

**Figure 1:**
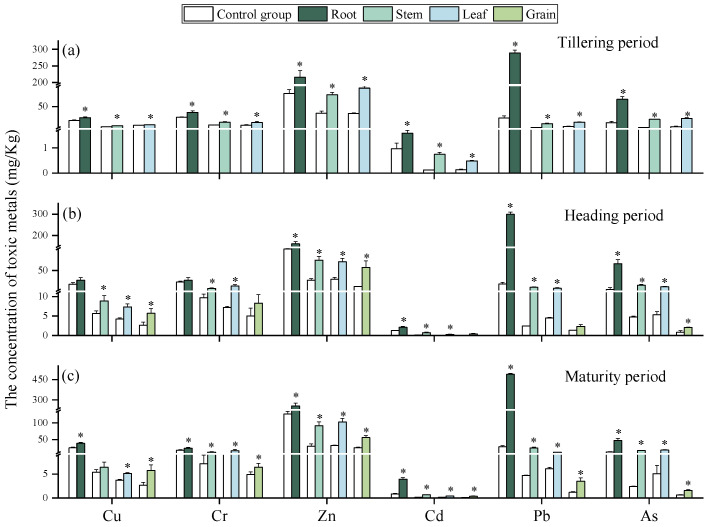
Content of HMs in the roots, stems, leaves, and grains of rice at tillering (**a**), heading (**b**), and maturity period (**c**). “*” expresses a significant difference between the control group and contaminated soil.

The contents of heavy metals (HMs) in different rice (*Oryza sativa* L.) tissues at three growth stages are shown in Figure 1. Compared with the control, HM contents in rice grown in contaminated soils were significantly higher. Across all stages, Cu, Cr, Zn, Cd, Pb, and As accumulated predominantly in roots, consistent with previous studies reporting higher HM levels in rice roots than in other tissues [27]. Root retention is considered a protective mechanism that limits HM translocation to stems, leaves, and grains [28]. As expected, HM contents was lowest in grains, aligning with earlier findings [15]. The distribution of HMs between stems and leaves varied with growth stage. At the heading stage, HM contents in stems exceeded those in leaves, likely due to the stem’s role as the primary transpiration channel, facilitating element transport during peak growth [18]. Specifically, at the tillering stage, Cr and Cd were higher in stems, while Cu, Zn, Pb, and As were higher in leaves. At maturity, Cu, Cd, and Pb accumulated more in stems, whereas Cr, Zn, and As were higher in leaves. For individual metals, Zn was the most abundant in stems, leaves, and grains across all stages, while Pb was consistently highest in roots. This pattern reflects soil contamination characteristics, as Pb contents in the tested soils were highest, followed by Zn (Table 1), indicating that soil quality strongly influences HM accumulation in crops [29].

#### 2.1.2. Bioaccumulation and Translocation Factors of HMs in Different Parts of Rice at Critical Growth Periods

The bioaccumulation and translocation factors (BF and TF) of HMs in the tested soils are presented in Table 2. The BFroot/soil values followed the order Cd > Cr > As > Zn ≈ Pb > Cu, Cd > Cu > Cr ≈ Pb > As > Zn, and Cd > Pb > Cu > Zn > Cr > As at the three growth stages, respectively. Cd consistently showed the highest BFroot/soil values across the rice growth cycle, which increased with time, reaching 1.95 at maturity. Similarly, the BFroot/soil values of Cu, Cd, and Pb increased with rice growth, while those of Cr and As decreased, indicating stage-dependent enrichment capacities. Previous studies also reported that metal enrichment capacity varies with growth stage, with Cd showing consistently higher root accumulation (BFroot/soil > 1 at heading and maturity), likely due to its dominance in exchangeable carbonate forms readily absorbed by root [30,31].

The TFstem/root values ranked Cd > Cr > Zn > As > Cu > Pb, Cr > Zn > As > Cd > Cu > Pb and Cr > As > Zn > Cd > Cu > Pb at the three stages, respectively. Cr exhibited the strongest transfer from roots to stems (except at tillering, when Cd was higher), while Pb consistently showed the weakest transfer. This pattern may be attributed to the essential role of Cr in plant metabolism versus the non-essential, passively absorbed nature of Pb [32,33]. The TFleaf/stem values followed Cu > Pb > Zn > As > Cr > Cd, Cr > Zn > Pb > As > Cu > Cd and Cr > Zn > As > Cu > Cd > Pb across the three stages. Similar to TFstem/root, Cr transfer to leaves was strongest at heading and maturity, while Cd transfer was weak, reflecting a protective mechanism restricting Cd translocation. The TFgrain/stem values ranked Zn > Cu > Cd > Cr > Pb > As at heading and Cu > Zn > Cd > Cr > Pb > As at maturity. The higher transfer of Zn and Cu to grains aligns with their role as essential nutrients, whereas Pb and As exhibited minimal transfer, consistent with their toxicity [34].

Overall, the BFroot/soil values of Cd were higher than the TF values, indicating that Cd primarily accumulated in roots with limited upward translocation. This is consistent with mechanisms such as phytochelatin chelation, vacuolar sequestration, and root surface adsorption [35]. In contrast, Cu, Cr, Zn, Pb, and As showed relatively higher TFleaf/stem values, suggesting effective upward transfer. μ-XRF imaging further confirmed the presence of As and Zn in roots, stems, leaves, and grains (Figure S1), supporting the conclusion that HMs absorbed by roots can be translocated to aerial parts of rice.

### 2.2. Toxicities of HMs on Rice Growth

#### 2.2.1. Effects of HMs on Rice Growth at Critical Growth Periods

**Figure 2:**
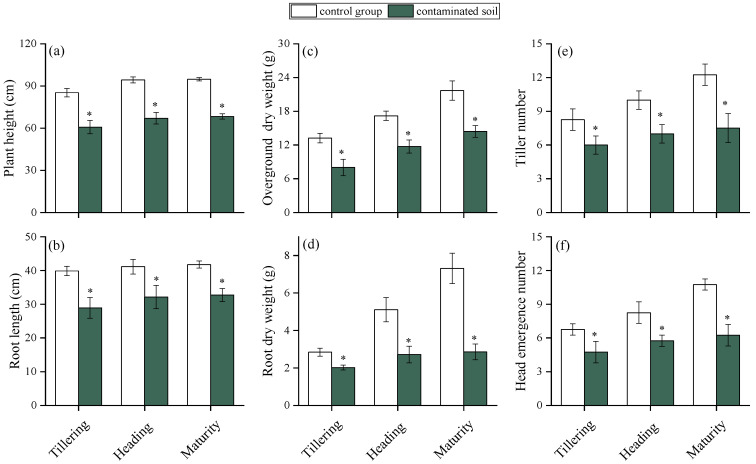
Effects of HMs on the plant height (**a**), root length (**b**), overground dry weight (**c**), root dry weight (**d**), tiller number (**e**), and head emergence number (**f**) of rice at different growth periods. “*” expresses a significant difference between the control group and contaminated soil.

The effects of HMs on rice growth at different stages are shown in Figure 2. Plant height, root length, aboveground and root dry weight, tiller number, and head emergence number were all significantly reduced in contaminated soils compared with the control (*p* < 0.05). HMs are known to impair essential physiological processes such as photosynthesis, respiration, and hormone regulation [36,37]. For example, Zn, Ni, Cd, and Cu have been reported to reduce maize root and leaf biomass [38], while Pb toxicity has been shown to suppress tillering, plant height, and biomass accumulation in rice [39]. In this study, rice plant height decreased by 31.4%, 28.8%, and 27.8%, and root length by 31.7%, 29.4%, and 21.6% at tillering, heading, and maturity stages, respectively. Aboveground dry weight declined by 44.7%, 27.1%, and 33.5%, while root dry weight decreased by 28.4%, 46.4%, and 60.6%. Tiller number was reduced by 31.7%, 33.2%, and 50.0%, and panicle emergence by 29.2%, 34.3%, and 42.0% across the three stages. Notably, the inhibitory effects on plant height, root length, and aboveground biomass lessened as rice grew, whereas reductions in root dry weight, tiller number, and panicle emergence became more pronounced. This divergence suggests that different rice organs vary in their sensitivity to HM stress, with reproductive and belowground traits more vulnerable at later growth stages.

#### 2.2.2. Effects of HMs on Antioxidant Defense System in Rice at Critical Growth Periods

**Figure 3:**
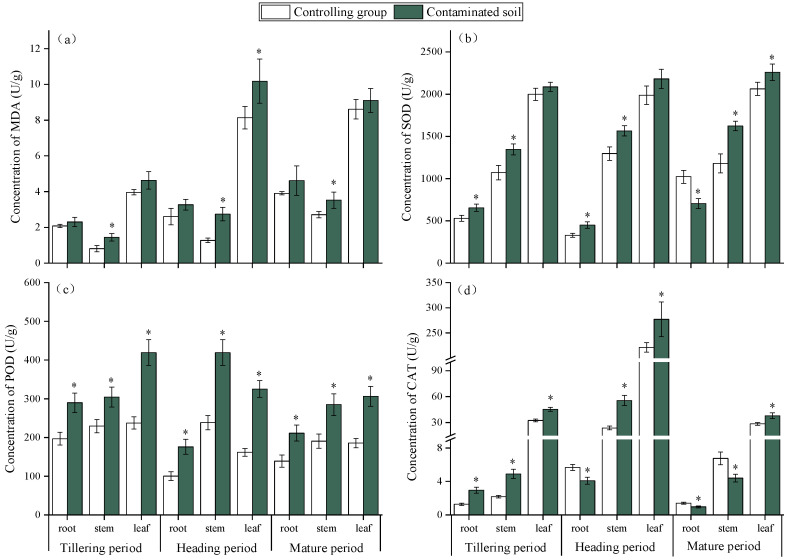
Effects of HMs on the MDA content (**a**), SOD activity (**b**), POD activity (**c**), and CAT activity (**d**) of rice at different growth periods. “*” expresses a significant difference between the control group and contaminated soil.

Heavy metal (HM) stress induces the excessive production of reactive oxygen species (ROS) in plants, leading to impaired cellular function and even cell death [40,41]. Malondialdehyde (MDA), a marker of lipid peroxidation, is widely used to assess oxidative damage. In this study, rice grown in HM-contaminated soil showed significantly higher MDA levels than the control (Figure 3a), confirming oxidative stress. Among tissues, MDA followed the order stems < roots < leaves, indicating that leaves were most sensitive. Compared with the control, MDA in roots increased by 11.0%, 28.9%, and 18.0% at the tillering, heading, and maturity stages, respectively; in stems, increases were 81.2%, 116.6%, and 30.4%; and in leaves, increases were 16.8%, 26.4%, and 6.3% at the three stages. Overall, MDA accumulation rose during early growth and declined later, reflecting initial sensitivity followed by enhanced tolerance to HMs [42].

To mitigate oxidative damage, plants activate antioxidant defense systems to maintain redox homeostasis, Superoxide dismutase (SOD), peroxidase (POD), and catalase (CAT) are key enzymes that scavenge ROS and reduce lipid peroxidation [43,44]. In this study, their activities in rice were generally higher under HM stress than in the control (Figure 3b–d), with leaves showing the strongest response, consistent with their higher MDA levels. Specifically, SOD activity increased in roots during tillering (24.3%) and heading (37.3%) but declined at maturity (−30.7%). In stems, SOD increased steadily (21–39%), while in leaves it rose moderately (4.6–10%). POD activity increased markedly across all tissues, particularly in leaves (76–101%). CAT activity showed sharp increases in roots (134%) and stems (126–136%) at early stages but decreased at maturity, whereas leaves maintained moderate increases (25–40%). These results demonstrate that HMs induce oxidative stress in rice, particularly in leaves, but plants counteract this damage through dynamic adjustments of antioxidant enzyme activity. The coordinated increase in SOD, POD, and CAT activity, especially at early stages, suggests an adaptive defense mechanism that alleviates ROS accumulation and reduces oxidative injury as rice growth progresses [45,46].

#### 2.2.3. Effect of HMs on Amino Acids Secretion in the Rhizosphere of Rice at Critical Growth Periods

**Figure 4:**
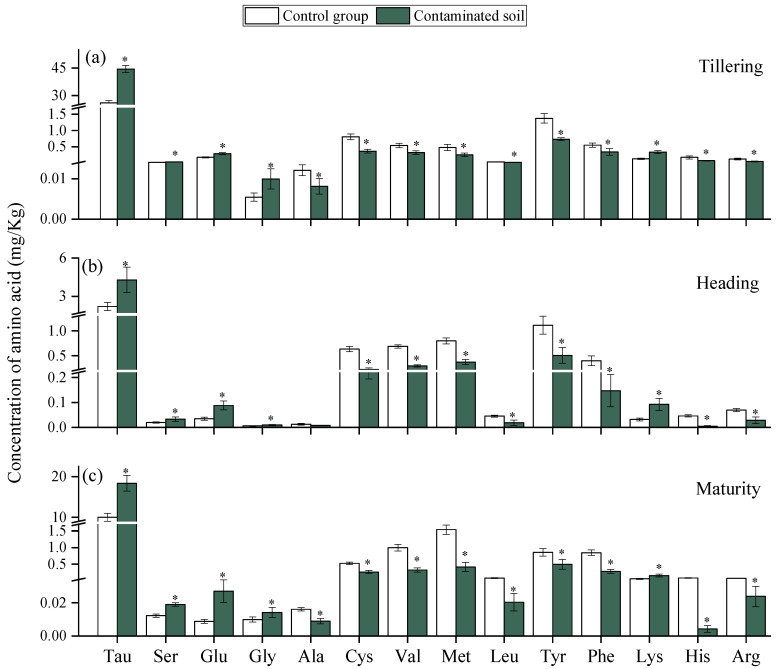
Effects of HMs on the amino acid in rice rhizosphere at Tillering (**a**), Heading (**b**), and Maturity (**c**) periods. “*” expresses a significant difference between the control group and contaminated soil.

Under heavy metal (HM) stress, plants adopt multiple defense mechanisms to alleviate damage, among which the secretion of root exudates plays a critical role [47]. In this study, the contents of Tau, Ser, Glu, Gly, and Lys in the tested soils were significantly higher than those in the control group at all three growth stages (Figure 4), indicating that HM stress stimulated amino acid secretion. Amino acids are known to participate in metal chelation, antioxidant defense, and signaling processes [48]. Amino acids can be complex with HMs, reducing their toxicity and organizing their entry into the plant. Their accumulation is considered as a positive response rather than the result of a metabolic disorder [47]. Previous studies also found that Cd stress changed the quantities and composition of amino acids in root exudates by the rice and increased the content of amino acids such as methionine, lysine, and histidine [49]. Among the amino acids, glutamic acid (Glu) exhibited the most pronounced response, with secretion levels increasing by 65.4%, 155.9%, and 211.8% compared with the control at the tillering, heading, and maturity stages, respectively. This progressive increase suggests that enhanced Glu secretion is a self-protective strategy of rice under HM stress. Previous research confirmed that Glu can alleviate Cd toxicity by inhibiting its uptake and translocation [50]. However, the contents of several amino acids, including Ala, Cys, Val, Met, Leu, Tyr, Phe, His, and Arg, were significantly reduced under HM stress. Similar reductions were observed in castor roots, where Cu exposure suppressed glutamic acid, alanine, cysteine, and methionine secretion [23]. It indicates that when HMs reach a specific content, they may inhibit the secretion of some amino acids by plant roots. And we found that the inhibitory effect of HMs stress on most amino acids increased with rice growth. This may be due to the accumulation of HMs in rice and the persistent effect on amino acid secretion in the rice root system.

### 2.3. Ecological Risk Assessment of HMs in Soil–Rice System

**Figure 5:**
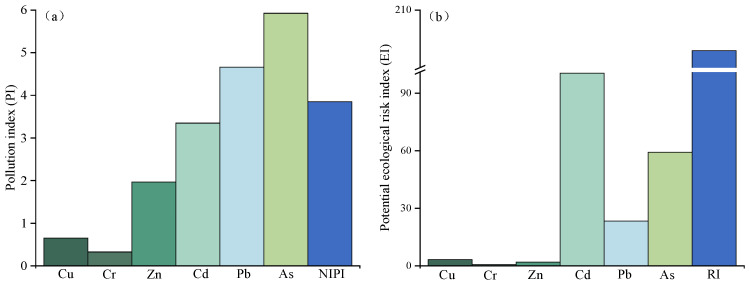
Pollution index (PI) (**a**) and Potential ecological risk index (EI) (**b**) of contaminated soil.

Assessment of HMs pollution in this study area were by PI, NIPI, EI, and RI. As shown in Table 1, the contents of Zn, Cd, Pb, and As in the tested soil significantly exceeded the Chinese soil quality risk control standard [51] (*p* < 0.05). As shown in Figure 5a, The NIPI values were all > 3 (Figure 5a), indicating that agricultural soils near the mine have reached a high pollution level, primarily due to HM-containing wastes released during lead-zinc mining and smelting [52]. Similar findings of elevated HMs in soils surrounding mining areas have been reported in other regions [53,54,55]. For individual metals, PI values followed the order As (5.92) > Pb (4.66) > Cd (3.35) > Zn (1.96) > Cu (0.65) > Cr (0.32). Thus, soils were mainly polluted by As, Pb, and Cd (PI > 3), while Zn showed low-level contamination (PI > 1). Previous studies in Pb-Zn mining areas, such as Guangxi, China, also identified Pb, Cd, and Zn as the dominant soil contaminants [56]. As contamination may be caused by the lead and zinc smelting process. The high As pollution observed here is likely linked to Pb-Zn smelting processes [57].

The ecological impact was further evaluated using EI and RI indices. The RI value indicates a moderate ecological risk (150 < RI < 300) (Figure 5b). Among individual metals, EI values ranked Cd (100.43) > As (59.24) > Pb (23.30) > Cu (3.26) > Zn (1.96) > Cr (0.65). Cd posed the greatest ecological threat, reaching a considerable risk level (80 < EI < 160), followed by As at a moderate level (40 < EI < 80). Pb, Cu, Zn, and Cr presented only low ecological risks (EI < 40). The dominant role of Cd in ecological risk reflects its high toxicity response factor compared with other metals [21,58]. This may result in the highest ecological risk from Cd.

### 2.4. Health Risk Assessment of HMs in Soil–Rice System

HMs in soils can pose serious health risks to humans through long-term exposure [59]. As shown in Table 3, the total hazard index (HI) values for adults and children were approximately 49 and 105 times higher than the non-carcinogenic risk threshold (>1). Similarly, the total carcinogenic risk (TCR) values were 423 and 892 times greater than the unacceptable level (>1 × 10^−4^), indicating that HMs contamination in agricultural soils near the mine represents severe non-carcinogenic and carcinogenic risks. Children were at higher risk than adults, likely due to higher exposure frequency, lower tolerance, and their developing physiological systems [60,61].

For both adults and children, exposure pathways ranked as food consumption > soil ingestion > soil inhalation > dermal absorption in terms of HI. For TCR, the order was food consumption > soil ingestion > soil inhalation > dermal absorption in adults, and food consumption > soil ingestion > dermal absorption > soil inhalation in children. Rice consumption was the dominant contributor to both non-carcinogenic and carcinogenic risks, consistent with previous finding [24,62]. As rice is a staple food, contamination may cause substantial health hazards. Notably, the HI from soil ingestion in children exceeded 1, highlighting that direct ingestion also poses significant non-carcinogenic risk for this group [63]. In contrast, soil inhalation and dermal absorption had relatively minor impacts.

For individual metals, non-carcinogenic risks exceeded the threshold (>1) for all HMs except Cu in adults, with Cr, As, and Pb as the major contributors. Regarding carcinogenic risks, TCR values from dermal absorption exceeded 1 × 10^−4^ for both adults and children, while soil ingestion contributed significantly for children and approached the threshold in adults. Among HMs, As posed the highest carcinogenic risk, followed by Cr, Cd, and Pb. Overall, As and Cr were the dominant contributors to both non-carcinogenic and carcinogenic risks, aligning with findings from other mining areas [64].

### 2.5. Health Risk Assessment by Monte Carlo Simulation

**Figure 6:**
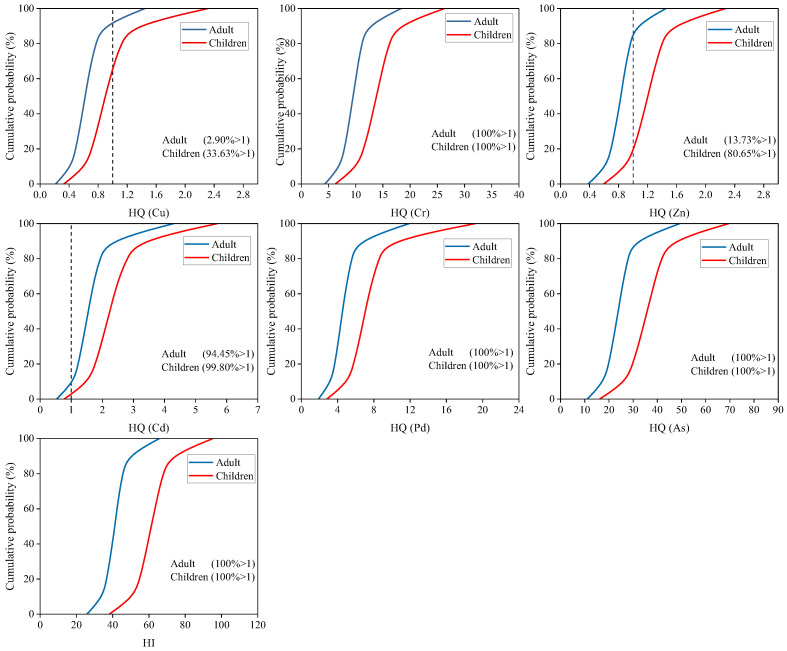
The probability distribution for the non-carcinogenic risk index of HMs.

**Figure 7:**
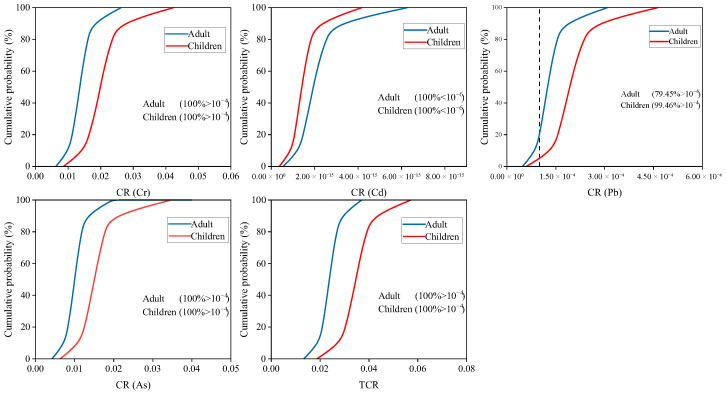
The probability distribution for carcinogenic risk index of HMs.

Assessment of uncertainty in non-carcinogenic and carcinogenic risk of HMs was by Monte Carlo simulation. As shown in Figure 6, 100% of HI values for adults and children exceeded the acceptable level (>1). For individual HMs, the non-carcinogenic risk of Cr, Pb and As showed a 100% probability of posing a risk to adults and children (HQ > 1). 99.5% (adults) and 99.8% (children) of HQ value from Cd were >1. The probability of non-carcinogenic risk from Cu for adults and children was 2.9% and 33.6%. Zn poses a low probability of non-carcinogenic risk to adults (13.7%) and a high non-carcinogenic risk to children (80.7%). As shown in Figure 7, the total carcinogenic risk for adults and children was 100% (TCR > 1 × 10^−4^). For individual HMs, the carcinogenic risk of Cr, Cd, and As showed a 100% probability of posing a risk to adults and children (CR > 1 × 10^−4^). And 79.5% (adults) and 99.5% (children) of CR value was found was for Pb > 1 × 10^−4^. It can be seen that the probability of Cr, Cd, Pb, and As causing non-carcinogenic and carcinogenic risks to residents was extremely high. Therefore, it is necessary to focus on monitoring the levels of these HMs in local soil and rice.

## 3. Materials and Methods

### 3.1. Study Area and Soil Sample Collection

The Qingchengzi polymetallic mining area (123°37′ E, 40°41′ N) is located in the northern region of Fengcheng City, northeast China. The site is approximately 120 km^2^, with an altitude of 250–500 m. This area belongs to the warm temperate continental monsoon climate zone, with an average annual temperature of 7.5 °C, average annual precipitation of 1000 mm, and an average annual frost-free period of 140 days. The primary parent rocks of the mine area are barite. The soil is brown loam, and the ore body is mainly a Pb-Zn mine.

**Figure 8:**
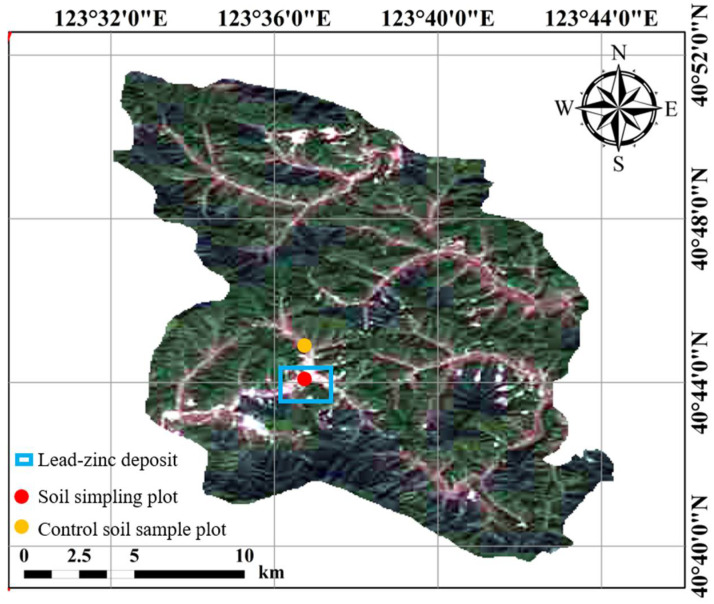
Location map of the study area.

Soil samples were collected from farmland surrounding the mining area using a plum–blossom sampling method. Five sampling points were selected, and the samples were composited (Figure 8). The control soil samples were collected from uncontaminated paddy soil using the same sampling method described above. The soil samples were air-dried and passed through a 2 mm sieve. Each pot was put into 1 kg of soil, and five seeds were sown in each pot. The rice (*Oryza sativa* L.) used in this study is Liaogeng-9, purchased from East Asia Seeds. The seeds were planted in inter-root bags and reduced to one plant after one week of emergence. The pot experiment followed local paddy management to simulate flooded conditions. Basal fertilizer was applied before sowing/transplanting (150 mg·N·kg^−1^, 75 mg·P_2_O_5_·kg^−1^, 100 mg·K_2_O·kg^−1^), with N topdressing at 50 mg·kg^−1^ at tillering and panicle initiation. Water depth was maintained at 2–3 cm (establishment), 5–7 cm (tillering), and 3–5 cm (heading to grain filling), with mid-season drainage for 5–7 days and final drainage 7–10 days before harvest; all treatments received the same water management. Each treatment was set up with four parallel replicates. All pots were placed outdoors on an open platform to simulate natural growth conditions. The pots were arranged in a completely randomized design, and their positions were randomly rotated weekly to minimize the effects of micro-environmental variation such as light exposure and wind direction.

The rice samples were collected at three key growth stages: tillering, heading, and maturity. The whole plants were taken out carefully with rhizosphere pockets. Rhizosphere soil tightly adhering to the rice root surface was carefully collected using a soft brush for the determination of amino acid contents. The rhizosphere soil samples were dried naturally, ground, and passed through a 100-mesh nylon sieve to measure HMs content. The rice samples were washed thoroughly with deionized water to measure plant height, root length, tiller number, head emergence number, and the dry weight of overground and root parts. After drying to constant weight in an 80 °C oven, rice was ground into powder and passed through an 100-mesh sieve. For biochemical analysis, washed fresh plant tissues were stored at −80 °C.

### 3.2. HMs Analysis in Rice and Soil Samples

#### 3.2.1. Determination of Heavy Metals (HMs) Concentrations in Rice and Soil

The powdered samples (0.5 g each) of different rice plant parts, including roots, stems, leaves, and grains, were digested in HNO_3_ and HClO_4_ (*v/v* = 10/3). The powdered samples (0.5 g each) of soil were digested in HNO_3_ and HClO_4_ (*v/v* = 12/7). Measurement of Cu, Cr, Zn, Cd, Pb, and As in plant and soil samples was performed using an inductively coupled plasma mass spectrometer (iCAP Q, Thermo Scientific, Waltham, MA, United States). The powdered samples (0.5 g each) of plant and soil were digested in NO_2_Cl (*m/v* = 1:1) for the measurement of As by atomic fluorescence spectrometer (AFS-85; Jitian Instruments Co., Ltd., Beijing, China).

#### 3.2.2. Analysis of HMs Distribution Characteristics in Rice

The method of the micro-X-ray fluorescence (μ-XRF) microspectroscopy experiment is shown in Text S1.

### 3.3. Ecotoxicological Testing of Rice Growth

#### 3.3.1. Determination of Antioxidant Enzyme System in Rice

The method for MDA content, SOD activities, POD activities, and CAT activities determination is described in Text S2.

#### 3.3.2. Determination of Amino Acids in Root Secretion of Rice

0.5 g of air-dried soil sample was loaded into a 100 mL hydrolysis bottle, 20 mL of HCL at a concentration of 6 mol/L was added, and it was 105 °C hydrolyzed for 24 h. The hydrolysate was filtered through Whatman 0.2 μm GD/X polyvinylidene fluoride membrane filter, dried by rotary evaporator (<45 °C), and redissolved with 4 mL of 0.05 mol/L HCl. The final purification was performed with polypropylene columns containing cation exchange resin. The solution pH was adjusted to 6.5–6.8 by adding NaOH and concentrated to 2 mL. The amino acid was detected by an amino acid analyzer (Hitachi, L8900, Tokyo, Japan). A total of 14 amino acids were detected, including taurine (Tau), serine (Ser), glutamic (Glu), glycine (Gly), alanine (Ala), cysteine (Cys), valine (Val), methionine (Met), leucine (Leu), tyrosine (Tyr), phenylalanine (Phe), lysine (Lys), histidine (His), and arginine (Arg).

### 3.4. Bioconcentration and Translocation Coefficients

The uptake capacity of rice for HMs in soil was evaluated by the bioconcentration factor (BF). The transfer ability of HMs in rice was evaluated by transfer factor (TF). The formula is as follows:

(1) $$\begin{array}{c} {\mathrm{BF}}_{\mathrm{root}/\mathrm{soil}}\;=\;\frac{C_{\mathrm{root}}}{C_{\mathrm{soil}}}\; \end{array}$$

(2) $$\begin{array}{c} {\mathrm{TF}}_{\mathrm{stem}/\mathrm{root}}=\frac{C_{\mathrm{stem}}}{C_{\mathrm{root}}}\; \end{array}$$

(3) $${\mathrm{TF}}_{\mathrm{leave}/\mathrm{stem}\;}=\frac{C_{\mathrm{leave}}}{C_{\mathrm{stem}}}\;$$

(4) $${\mathrm{TF}}_{\mathrm{grain}/\mathrm{stem}}=\frac{C_{\mathrm{grain}}}{C_{\mathrm{stem}}}$$

where *C_soil_* refer to the content of HMs in the soil (mg/kg DW). *C_root_*, *C_stem_*, *C_leave_*, and *C_grain_* refer to the content of HMs in rice roots, stems, leaves, and grains, respectively (mg/kg DW).

### 3.5. Nemerow Pollution Index and Potential Ecological Risk Assessment of Soil System

The Nemerow pollution index (NIPI) was used to assess soil HMs contamination; the formula is as follows:

(5) $$\mathrm{PI}\;=\;\frac{C_{i}}{S_{i}}$$

(6) $$\begin{array}{c} NIPI=\sqrt{\frac{{\left(PI_{i}\right)}_{\mathrm{av}\;\mathrm{e}}^{2}+{\;\left(PI_{i}\right)}_{\max}^{2}}{2}} \end{array}$$

where *PI* is the pollution index of individual HMs, *C_i_* refers to the content of i element in soil sample, and *S_i_* refers to the soil quality standard. *(PI_i_)_ave_* refers to the average of *PI* values for HMs, and *(PI_i_)_max_* refers to the maximum PI value of HMs.

The potential ecological risk index (RI) quantitatively assesses the possible ecological risk caused by HMs considering the content of each heavy metal in the environment, its ecological effect, and the difference in its toxicity to the environment [65]. The formula is as follows:

(7) $$\begin{array}{c} EI\;=\;T_{i}\;\times \;\frac{C_{i}}{S_{i}} \end{array}$$

(8) $$\begin{array}{c} RI=\sum_{i=1}^{n}EI \end{array}$$

where *EI* is the potential ecological index of individual HMs and *T_i_* is toxic response factor. The values of the above parameters are shown in Table S1. The classification of *PI*, NIPI, *EI*, and *RI* are shown in Tables S2 and S3.

### 3.6. Health Risk Assessment

HMs in soils and crops cause potential health risks to human health in two main ways: long-term exposure to contaminated soil particles and food consumption of contaminated crops. Ingestion, inhalation, and dermal contact are the main ways humans are exposed to HMs in soil. This paper evaluates human risk through the non-carcinogenic risk index (HI) and carcinogenic risk index (CR). For non-carcinogenic, the potential ecological risk of individual HMs can be expressed by hazard quotients (HQ), and the total potential health risk is expressed by hazard index (HI) [66]. The formula is as follows:

(9) $$\begin{array}{c} {\mathrm{ADI}}_{\mathrm{food}}\;=\;\frac{C_{\mathrm{rice}}\;\times \;\mathrm{IR}\;\times \;\mathrm{EF}\;\times \;\mathrm{ED}}{\mathrm{BW}\;\times \;\mathrm{AT}} \end{array}$$

(10) $$\begin{array}{c} {\mathrm{ADI}}_{\mathrm{ing}}=\frac{C_{\mathrm{soil}}\;\times \;\mathrm{IngR}\;\times \;\mathrm{EF}\;\times \;\mathrm{ED}}{\mathrm{BW}\;\times \;\mathrm{AT}}\;\times {\;10}^{-6} \end{array}$$

(11) $$\begin{array}{c} {\mathrm{ADI}}_{\mathrm{inh}}=\frac{C_{\mathrm{soil}}\;\times \;\mathrm{InhR}\;\times \;\mathrm{EF}\;\times \;\mathrm{ED}}{\mathrm{PEF}\;\times \;\mathrm{BW}\;\times \;\mathrm{AT}}\;\times {\;10}^{-6} \end{array}$$

(12) $$\begin{array}{c} {\mathrm{ADI}}_{\mathrm{der}}=\frac{C_{\mathrm{soil}}\;\times \;\mathrm{SA}\;\times \;\mathrm{AF}\;\times \;\mathrm{ABS}\;\times \;\mathrm{EF}\;\times \;\mathrm{ED}}{\mathrm{BW}\;\times \;\mathrm{AT}}\;\times {\;10}^{-6} \end{array}$$

where *ADI_food_*, *ADI_ing_*, *ADI_inh_*, and *ADI_der_* are the average daily intake through rice, soil ingestion, inhalation, and dermal absorption (mg/kg/day). *C_rice_* and *C_soil_* are the HMs content in rice and soil (mg/kg). The meanings and values of each of the above parameters are shown in Table S4.

The non-carcinogenic risk of HMs can be calculated using the following formula:

(13) $$\begin{array}{c} HQ\;=\;\frac{\mathrm{ADI}}{\mathrm{RFD}} \end{array}$$

(14) $$\begin{array}{c} HI=\sum_{i=1}^{n}\mathrm{HQ} \end{array}$$

where *HQ* is the assessed non-carcinogenic health risk of individual HMs and *HI* is used to estimate the overall non-carcinogenic health risk. *RFD* is the reference dose of each heavy metal (mg/kg/day). The *HQ* and *HI* values > 1 indicate that non-carcinogenic effects may occur, and the values < 1 indicate no potential risk [21,60].

The carcinogenic risk of HMs can be calculated using the following formula:

(15) $$\begin{array}{c} CR\;=\;ADI\;\times \;SF \end{array}$$

(16) $$\begin{array}{c} TCR=\sum_{i=1}^{n}\mathrm{CR} \end{array}$$

where *CR* is the assessed carcinogenic health risk of individual HMs and *TCR* is the total carcinogenic risk. *SF* is the slope factor of HMs. The *CR* and *TCR* values >1 × 10^−4^ indicate an unacceptable risk; the values between 1 × 10^−4^ and 1 × 10^−6^ indicate an acceptable risk; the values < 1 × 10^−6^ indicate no obvious risk [21]. The values of *RFD* and *SF* are shown in Table S5.

### 3.7. Monte Carlo Simulation

Monte Carlo simulation can estimate the probability distribution of health risks by calculating the relevant variables of the input [25]. To more accurately assess the health risks associated with HMs, uncertainties in HM concentrations, oral intake, inhalation and body weight, and exposure time were considered. The Monte Carlo simulation was performed using Oracle Crystal Ball software (v11.1.2.4) for 10,000 calculations. The values of parameters used in the Monte Carlo simulation are shown in Table S6.

### 3.8. Statistical Analysis

In this study, Excel (v2019) was used to process data. SPSS (v23) was used to analyze differences among treatments using one-way ANOVA and Tukey’s HSD test, and homogeneity of variance was checked for each dataset. Each mean was based on four replicates, and error bars represent the standard deviation (SD). Differences were considered statistically significant at *p* < 0.05. Origin pro (v2023) was used to draw related charts in the article.

## 4. Conclusions

Our study demonstrated that heavy metals (HMs) contents in rice (*Oryza sativa* L.) fields near the Pb-Zn mine was significantly higher than those in the control. In rice, HMs were mainly retained in roots and least in grains across all growth stages. The enrichment and translocation capacities of rice varied with development, but Cd consistently showed the highest enrichment and was readily transferred from roots to aboveground parts. HM stress significantly inhibited rice growth, reducing plant height, root length, biomass, tiller number, and panicle emergence. It also induced oxidative damage, as indicated by increased MDA levels, while activating antioxidant defenses (SOD, POD, CAT). In addition, rice mitigated HM stress by modulating root amino acid exudation. Soil in the mining area was severely contaminated with As, Pb, and Cd as the dominant pollutants, followed by Zn. The potential ecological risk was moderate, but Cd posed considerable risk due to its high toxicity. Health risk assessment revealed serious non-carcinogenic and carcinogenic risks to residents, with children more vulnerable than adults. Monte Carlo simulation results indicate that HMs in soil pose a 100% total non-carcinogenic and carcinogenic risk for residents, with Cr, Cd, Pb, and As posing particularly high risks. In summary, contaminated soils near mining areas are not suitable for crop cultivation, especially for rice.

## Figures and Tables

**Table 1:** Physical and chemical properties of soil.

Physiochemical Properties	Contaminated Soil	Control Soil
pH	6.15 ± 0.02	6.22 ± 0.01
SOC (g/kg)	10.74 ± 0.45	10.55 ± 2.87
TN (g/kg)	1.48 ± 0.06	1.39 ± 0.07
TK (g/kg)	18.87 ± 0.08	19.01 ± 0.02
Cu (mg/kg)	65.18 ± 4.03 *	23.98 ± 1.43
Cr (mg/kg)	69.75 ± 2.96 *	38.46 ± 2.24
Zn (mg/kg)	490.87 ± 15.84 *	123.94 ± 10.59
Cd (mg/kg)	2.01 ± 0.29 *	0.27 ± 0.03
Pb (mg/kg)	652.34 ± 29.73 *	31.92 ± 3.45
As (mg/kg)	148.11 ± 8.07 *	13.08 ± 1.73

Note: “*” expresses a significant difference between the control group and contaminated soil.

**Table 2:** BFs and TFs values of rice for HMs.

Heavy Metal (mg/kg)	Growth Period	BF_root/soil_	TF_stem/root_	TF_leave/stem_	TF_grain/stem_
Cu	Tillering	0.38 ± 0.03 b	0.27 ± 0.04 a	1.34 ± 0.14 a	—
	Heading	0.49 ± 0.07 ab	0.28 ± 0.04 a	0.84 ± 0.22 b	0.66 ± 0.20 a
	Maturity	0.60 ± 0.04 a	0.16 ± 0.03 b	0.81 ± 0.11 b	0.91 ± 0.26 a
Cr	Tillering	0.53 ± 0.05 a	0.41 ± 0.05 a	0.95 ± 0.12 a	—
	Heading	0.46 ± 0.07 ab	0.52 ± 0.09 a	1.28 ± 0.19 a	0.51 ± 0.15 a
	Maturity	0.35 ± 0.04 b	0.53 ± 0.05 a	1.35 ± 0.46 a	0.50 ± 0.05 a
Zn	Tillering	0.44 ± 0.04 b	0.36 ± 0.02 b	1.20 ± 0.13 a	—
	Heading	0.33 ± 0.02 c	0.43 ± 0.02 a	0.97 ± 0.19 a	0.80 ± 0.11 a
	Maturity	0.52 ± 0.04 a	0.36 ± 0.03 b	1.14 ± 0.24 a	0.61 ± 0.01 a
Cd	Tillering	0.79 ± 0.06 c	0.47 ± 0.06 a	0.65 ± 0.03 a	—
	Heading	1.04 ± 0.13 b	0.35 ± 0.10 a	0.44 ± 0.14 a	0.52 ± 0.09 a
	Maturity	1.95 ± 0.16 a	0.17 ± 0.00 b	0.61 ± 0.13 a	0.55 ± 0.11 a
Pb	Tillering	0.44 ± 0.01 b	0.04 ± 0.00 b	1.31 ± 0.20 a	—
	Heading	0.46 ± 0.02 b	0.06 ± 0.00 a	0.90 ± 0.03 b	0.13 ± 0.02 a
	Maturity	0.75 ± 0.01 a	0.05 ± 0.01 a	0.51 ± 0.04 c	0.14 ± 0.04 a
As	Tillering	0.45 ± 0.04 a	0.33 ± 0.04 a	1.09 ± 0.05 a	—
	Heading	0.42 ± 0.05 ab	0.36 ± 0.05 a	0.87 ± 0.10 b	0.09 ± 0.01 a
	Maturity	0.32 ± 0.05 b	0.38 ± 0.06 a	1.07 ± 0.08 a	0.09 ± 0.01 a

Note: Different letters indicate significant differences in the bioaccumulation and translocation factors of HMs in rice among different growth stages (*p* < 0.05).

**Table 3:** The non-carcinogenic risk assessment of HMs.

Heavy Metal		HQ_food_	HQ_ing_	HQ_inh_	HQ_der_	Total	CR_food_	CR_ing_	CR_inh_	CR_der_	Total
Cu	adults	7.72 × 10^−1^	5.21 × 10^−4^	3.05 × 10^−14^	3.46 × 10^−5^	7.73 × 10^−1^					
	children	1.61	3.19 × 10^−3^	3.55 × 10^−14^	1.19 × 10^−4^	1.62					
Cr	adults	11.63	7.43 × 10^−3^	4.59 × 10^−10^	1.48 × 10^−4^	11.64	1.74 × 10^−2^	1.11 × 10^−5^	5.51 × 10^−13^	8.90 × 10^−6^	1.74 × 10^−2^
	children	24.38	4.55 × 10^−2^	5.33 × 10^−10^	5.10 × 10^−4^	24.43	3.66 × 10^−2^	6.82 × 10^−5^	6.41 × 10^−13^	3.06 × 10^−5^	3.67 × 10^−2^
Zn	adults	1.01	5.23 × 10^−4^	3.08 × 10^−13^	5.22 × 10^−5^	1.01					
	children	2.12	3.20 × 10^−3^	3.58 × 10^−13^	1.79 × 10^−4^	2.12					
Cd	adults	1.93	6.42 × 10^−4^	3.78 × 10^−13^	1.28 × 10^−3^	1.93	1.18 × 10^−2^	3.92 × 10^−6^	2.38 × 10^−15^	7.81 × 10^−8^	1.18 × 10^−2^
	children	4.05	3.93 × 10^−3^	4.39 × 10^−13^	4.40 × 10^−3^	4.06	2.47 × 10^−2^	2.40 × 10^−5^	2.77 × 10^−15^	2.69 × 10^−7^	2.47 × 10^−2^
Pb	adults	5.38	5.96 × 10^−2^	3.48 × 10^−11^	7.92 × 10^−3^	5.45	1.60 × 10^−4^	1.77 × 10^−6^	5.15 × 10^−15^	—	1.62 × 10^−4^
	children	11.27	3.65 × 10^−1^	4.05 × 10^−11^	2.72 × 10^−2^	11.67	3.35 × 10^−4^	1.09 × 10^−5^	5.99 × 10^−15^		3.46 × 10^−4^
As	adults	28.23	1.58 × 10^−1^	2.26 × 10^−10^	2.30 × 10^−1^	28.61	1.27 × 10^−2^	7.10 × 10^−5^	4.20 × 10^−13^	1.04 × 10^−4^	1.29 × 10^−2^
	children	59.18	9.66 × 10^−1^	2.63 × 10^−10^	7.92 × 10^−1^	60.94	2.66 × 10^−2^	4.35 × 10^−4^	4.89 × 10^−13^	3.56 × 10^−4^	2.74 × 10^−2^
HI/TCR	adults	48.95	2.26 × 10^−1^	7.21 × 10^−10^	2.41 × 10^−1^	49.41	4.21 × 10^−2^	8.78 × 10^−5^	9.79 × 10^−13^	1.13 × 10^−4^	4.23 × 10^−2^
	children	102.62	1.39	8.38 × 10^−10^	8.24 × 10^−1^	104.83	8.82 × 10^−2^	5.38 × 10^−4^	1.14 × 10^−12^	3.87 × 10^−4^	8.92 × 10^−2^

## Data Availability

Data are contained within the article.

## Supplementary Materials

The following supporting information can be downloaded at: https://www.mdpi.com/article/10.3390/plants15010030/s1, Text S1. Analysis of HMs distribution characteristics in rice; TextS2. The method for MDA content, SOD activities, POD activities and CAT activities determination; Table S1. Pollution index of HMs in soil; Table S2. The classes of index (PI) and Nemerow integrated pollution index (NIPI); Table S3. The classes of Single Potential ecological risk level (EI) and Potential ecological risk level (RI); Table S4. The value of exposure indices of human health risk assessment; Table S5. Values of reference dose (RfD, mg/kg/d) and slope factor (SF, mg/kg/d) for elements; Table S6. The value of parameters used in the Monte-Carlo simulation model; Figure S1. μ-XRF imaging of different part of rice. Grain (a,b), leave (c,d), rootstock union (e,f), root profile (g–i). Higher fluorescence intensities (corresponding to higher concentrations) are nearer to red and lower intensities are nearer to blue according to the color bars. Refs. [8,21,65,66,67,68,69,70,71] have been cited in the supplementary materials.
