# Supplementary material for: Ecotoxicological Effects of Heavy Metals on Rice (Oryza sativa L.) Across Its Life Cycle and Health Risk Assessment in Soils Around Pb–Zn Mine
Source: Plants (Basel). 2025 Dec 21;15(1):30. doi: 10.3390/plants15010030 (PMC12787725; doi:10.3390/plants15010030)
Supplement: Supplementary file 1 [file plants-15-00030-s001.zip › plants-4033780-supplementary.pdf]

Ecotoxicological effects of heavy metals on rice across its life cycle and health risk assessment in agricultural soils around Pb–Zn mine

Fangyu Hu[1,a], Baoyu Wang[1,2,a], Lingyan Zhang[1], Yue Wang[1,2], Jiaqi Sha[1,2], Jinhao Dong[1,2], Hewei Song[1], Jing An[1*]

1. Institute of Applied Ecology, Chinese Academy of Sciences, Shenyang 110016, China;
2. University of Chinese Academy of Sciences, Beijing 100049, China;
3. School of Municipal and Environmental Engineering, Shenyang Jianzhu University, Shenyang, Liaoning 110168, China
a. Equal contributor.

List of Supplementary Information

Text S1 Analysis of HMs distribution characteristics in rice

TextS2 The method for MDA content, SOD activities, POD activities and CAT activities determination

Table S1 Pollution index of HMs in soil.

Table S2 The classes of index (PI) and Nemerow integrated pollution index (NIPI).

Table S3 The classes of Single Potential ecological risk level (EI) and Potential ecological risk level (RI)

Table S4 The value of exposure indices of human health risk assessment

Table S5 Values of reference dose (RfD, mg/kg/d) and slope factor (SF, mg/kg/d) for elements.

Table S6 The value of parameters used in the Monte-Carlo simulation model

Fig. S1. μ-XRF imaging of different part of rice. Grain (a-b), leave (c-d), rootstock union (e-f), root profile (g-i). Higher fluorescence intensities (corresponding to higher concentrations) are nearer to red and lower intensities are nearer to blue according to the color bars.

TextS1 Analysis of HMs distribution characteristics in rice

The samples were collected from rice plants at the maturity stage of the pot experiment. One representative plant was selected from each treatment for μ-XRF analysis. Cross-sections of roots, root–stem junctions, leaves, and grains were scanned. Fresh rice tissues were transversely sectioned into slices with a thickness of 20 μm. The sections were mounted onto XRF tape and fixed on sample holders for scanning. A total of 9 samples were prepared.

The 4W1B endstation of Beijing synchrotron Radiation Facility is to perform the micro-X-ray fluorescence (μ-XRF) microspectroscopy experiment, which runs 2.5 GeV electron with current from 150 mA to 250 mA. The incident X-ray energy at 15 keV is monochromatized by W/B4C Double-Multilayer-Monochromator (DMM) and the polycapillary lens is used to focus down to 50 μm in diameter. Then the sample is held on a precision motor-driven stage and scanning 50 μm stepwise, to gain two-dimensional mapping. The Si (Li) solid state detector is used to detect X-ray fluorescence emission lines with live time of 10 s. PyMCA package is uesd to reduce and process the data.

TextS2 The method for MDA content, SOD activities, POD activities and CAT activities determination

Malondialdehyde (MDA) content was determined using the thiobarbituric acid (TBA) method[1]. Fresh leaf tissue (0.2 g) was homogenized with 5 mL of 10% (w/v) trichloroacetic acid (TCA) and a small amount of quartz sand, transferred to a 15 mL centrifuge tube, brought to volume, and centrifuged at 3000 g for 10 min; the supernatant was used as the extract. An aliquot of 2 mL extract was mixed with 2 mL of 0.6% TBA solution, sealed, heated in a boiling water bath for 15 min, rapidly cooled, and centrifuged again, after which absorbance was measured at 600, 532, and 450 nm, with 2 mL distilled water replacing the extract as the blank.

For crude enzyme extraction, 0.2 g of fresh rice root, stem, or leaf tissue was ground in a pre-cooled mortar with 5 mL pre-cooled 0.05 mol L^{-1} phosphate buffer (pH 7.80) and quartz sand on ice, transferred to a 15 mL tube, brought to volume, and centrifuged at 10,000 g for 10 min at 4 °C; the supernatant was collected as the crude enzyme extract and stored at 4 °C. Superoxide dismutase (SOD) activity was assayed by the nitro blue tetrazolium (NBT) photoreduction method by incubating 100 μL crude enzyme extract with 3 mL SOD reaction solution in transparent tubes under 4000 lux light at 25 ± 2 °C for 20 min; phosphate buffer (pH 7.80) was used instead of enzyme extract for controls, with one control kept in the dark for instrument zeroing and another exposed to light to obtain the maximum photoreduction value; absorbance was then recorded at 560 nm[2]. Catalase (CAT) activity was measured using the UV absorption method by adding 50 μL crude enzyme extract to 3 mL CAT reaction solution, mixing rapidly, and recording the change in absorbance at 240 nm within 1 min, using 50 μL phosphate buffer (pH 7.00) as the blank[3]. Peroxidase (POD) activity was determined using the guaiacol method by adding 20 μL crude

enzyme extract to 3 mL POD reaction solution and recording the change in absorbance at 470 nm within 1 min, with 20 μL phosphate buffer (pH 6.0) substituted for the enzyme extract as the blank[4].

Table S1 Pollution index of HMs in soil.

Heavy metal (mg/kg)	GB15618-2018	T_i
Cu	100	5
Cr	300	2
Zn	25	1
Cd	0.6	30
Pb	140	5
As	25	10

"*" expresses a significant difference between the control group and contaminated soil.

Table S2 The classes of index (PI) and Nemerow integrated pollution index (NIPI).

PI	Soil quality	NIPI	Soil quality
PI ≤ 1	Non-contaminated	NIPI ≤ 0.7	Non-contaminated
1 < PI ≤ 2	Low level of pollution	0.7 < NIPI ≤ 1	Warning line of pollution
2 < PI ≤ 3	Moderate level of pollution	1 < NIPI ≤ 2	Slightly to moderately contaminated
PI > 3	High level of pollution	2 < NIPI ≤ 3	Moderately contaminated
		NIPI > 3	High level of pollution

Table S3 The classes of Single Potential ecological risk level (EI) and Potential ecological risk level (RI)

EI	Single Potential ecological risk level	RI	Potential ecological risk level
EI < 40	Low risk	RI < 150	Low risk
40 < EI < 80	Moderate risk	150 < RI < 300	Moderate risk
80 < EI < 160	Considerable risk	300 < RI < 600	Considerable risk
160 < EI < 320	High risk	≥ 600	Very high risk
≥ 320	Very high risk		

Table S4 The value of exposure indices of human health risk assessment

Parameter	Decsription	Unit	Adult	Child	Reference
IR	Intake rate of rice	kg/d	0.3367	0.2885	[7]<br>[7]
IngR	Ingestion rate of soil	mg/d	20	50	[7]
InhR	Inhalation rate of soil	m^3/d	16	7.6	[7]
					[5]
EF	Exposure frequency	d/a	350	350	[7]
ED	Exposure duration (Non-carcinogens)	a	24	6	[7]
ED	Exposure duration (carcinogens)	a	70	70	[7]<br>[5]
SA	Exposed area through dermal contact	cm^2	5700	2800	[7]
AF	adherence factor	mg/cm^2/d	0.07	0.2	[7]
ABS	Dermal absorption factor		As (0.03) other (0.001)	As (0.03) other (0.001)	[7]
BW	Average body weight	kg	60.1	24.5	[7]
AT	Average exposure time	d	ED×365	ED×365	[7]
PEF	Particle emission factor	m^3/kg	1.36×10^9	1.36×10^9	[7]

Table S5 Values of reference dose (RFD, mg/kg/d) and slope factor (SF, mg/kg/d) for elements[5,6].

Heavy metal	RFD			SF		
	Ingest	Inhale	Dermal	Ingest	Inhale	Dermal
Cu	0.04	0.0402	0.012			
Cr	0.003	0.0000286	0.003	0.5	42	20
Zn	0.300	0.300	0.060			
Cd	0.001	0.001	0.00001	6.1	6.3	6.1
Pb	0.0035	0.00352	0.000525	0.0085	0.042	
As	0.0003	0.000123	0.000123	1.5	15.1	3.66

Table S6 The value of parameters used in the Monte-Carlo simulation model

Parameter	Unit	Probabilistic distribution	Value	Reference
C	mg/kg	Log-normal	This study	[7]
IR	mg/d	Triangular (adults)	TRI (0.25, 0.2885, 0.30)	[7]
		Triangular (children)	TRI (0.28, 0.3367, 0.35)	[5]
IngR	mg/d	Triangular (adults)	TRI (66, 103, 161)	[7]
		Triangular (children)	TRI (4, 30, 52)	[7]
InhR	m^3/d	Log-normal (adults)	LN (7.19,1.62)	[5]
		Log-normal (children)	LN (16.57,4.05)	[7]
BW	kg	Uniform (adults)	UN (55.7, 68.6)	[7]
		Log-normal (children)	LN (37.0, 2.98)	[7]
EF	d/a	Triangular	TRI (180, 345, 365)	[5]
ED	a	Point	6(children), 24(adults)	[7]
AT	d	Point	ED×365	[7]
SA	cm^2	Point	5700(adults), 2800(children)	[7]
AF		Point	0.2(adults), 0.07(children)	[7]
ABS		Point	0.03 (As), 0.001 (others)	[7]
Rfd	mg/kg/d	Point		[7]
SF	mg/kg/d	Point		[7]

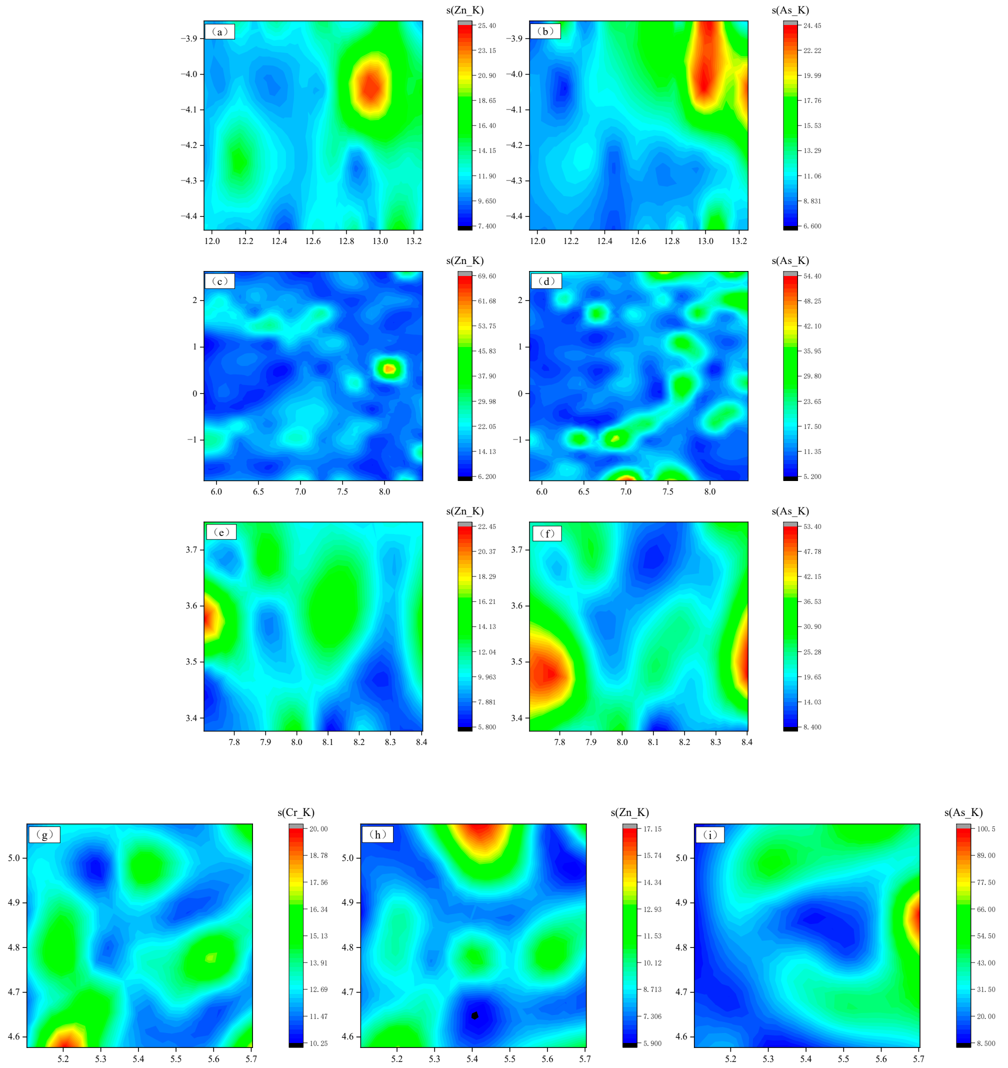

Fig. S1. μ-XRF imaging of different part of rice. Grain (a-b), leave (c-d), rootstock union (e-f), root profile (g-i). Higher fluorescence intensities (corresponding to higher concentrations) are nearer to red and lower intensities are nearer to blue according to the color bars.

Reference

[1]Beauchamp, C., Fridovich, I., 1971. Superoxide dismutase: improved assays and an assay applicable to acrylamide gels. Anal. Biochem. 44, 276–287.

[2]Beer Jr., R.F., Sizer, I.W., 1952. A spectrophotometric method for measuring the breakdown of hydrogen peroxide by catalase. J. Biol. Chem. 195, 133–140.

[3]Zhang, J., Cui, S., Li, J., Kirkham, M.B., 1995. Protoplasmic factors, antoxidant responses, and chilling resistance in maize. Plant Physiol. Biochem. 33, 567–575.

[4]Kosugi, H., Kikugawa, K., 1985. Thiobarbituric acid reaction of aldehydes and oxidized lipids in glacial acetic acid. Lipids 20, 915–920.

[5]Chen L., .Zhou M., .Wang J., .Zhang Z., .Duan C., .Wang X., .Zhao S., .Bai X., .Li Z., .Li Z., .Fang L., 2022. A global meta-analysis of heavy metal(loid)s pollution in soils near copper mines: Evaluation of pollution level and probabilistic health risks. Sci Total Environ, 835, 155441.

[6]Kan X., .Dong Y., .Feng L., .Zhou M., .Hou H., 2021. Contamination and health risk assessment of heavy metals in China’s lead–zinc mine tailings: A meta–analysis. Chemosphere, 267.

[7]USEPA (United States Environmental Protection Agency), 2011. Risk Based Concentration Table. United States Environmental Protection Agency, Washington DC.
